# Protein import into chloroplasts and its regulation by the ubiquitin-proteasome system

**DOI:** 10.1042/BST20190274

**Published:** 2020-01-10

**Authors:** Simon M. Thomson, Pablo Pulido, R. Paul Jarvis

**Affiliations:** Department of Plant Sciences, University of Oxford, South Parks Road, Oxford OX1 3RB, U.K.

**Keywords:** chloroplasts, organelle biogenesis, plastid, protein import, protein translocation, ubiquitin-proteasome system

## Abstract

Chloroplasts are photosynthetic plant organelles descended from a bacterial ancestor. The vast majority of chloroplast proteins are synthesized in the cytosol and then imported into the chloroplast post-translationally. Translocation complexes exist in the organelle's outer and inner envelope membranes (termed TOC and TIC, respectively) to facilitate protein import. These systems recognize chloroplast precursor proteins and mediate their import in an energy-dependent manner. However, many unanswered questions remain regarding mechanistic details of the import process and the participation and functions of individual components; for example, the cytosolic events that mediate protein delivery to chloroplasts, the composition of the TIC apparatus, and the nature of the protein import motor all require resolution. The flux of proteins through TOC and TIC varies greatly throughout development and in response to specific environmental cues. The import process is, therefore, tightly regulated, and it has emerged that the ubiquitin-proteasome system (UPS) plays a key role in this regard, acting at several different steps in the process. The UPS is involved in: the selective degradation of transcription factors that co-ordinate the expression of chloroplast precursor proteins; the removal of unimported chloroplast precursor proteins in the cytosol; the inhibition of chloroplast biogenesis pre-germination; and the reconfiguration of the TOC apparatus in response to developmental and environmental signals in a process termed chloroplast-associated protein degradation. In this review, we highlight recent advances in our understanding of protein import into chloroplasts and how this process is regulated by the UPS.

## Introduction

Chloroplasts are organelles found within plants and algae which house a range of essential metabolic and biosynthetic functions, including photosynthesis [1,2]. Chloroplasts are endosymbiotically derived organelles, and consequently, contain a functional genome interpreted by eubacterial-type transcription and translation machineries, and possess a double-membraned envelope [3]. Over the course of plant evolution, roughly 98% of the endosymbiont's protein-coding sequences were either lost or relocated to the nuclear genome by the process of endosymbiotic gene transfer [4]. Approximately 100 genes are retained in the chloroplast, and these encode vital factors such as core genetic components [5]. The remaining 2000–3000 chloroplast proteins are encoded in the nucleus and imported post-translationally from the cytosol [6–8]. Chloroplasts are fully integrated cellular components that participate in bidirectional communication with the nucleus to co-ordinate gene expression for organelle biogenesis, photosynthesis, and plastid metabolism [9–11].

Chloroplast-localized proteins are typically synthesized as precursors, each with an N-terminal targeting signal called a transit peptide (TP), and delivered to the chloroplast surface by cytosolic chaperones. Entry into the chloroplast is achieved via translocon complexes in the outer (TOC) and inner (TIC) envelope membranes, which recognize the TP and facilitate transport into the stroma. The TPs of translocated precursor proteins (pre-proteins) are then cleaved off by the stromal processing peptidase (SPP) [12], before final folding and assembly in the stroma, or onward routing to the thylakoids [13,14] or the inner envelope membrane (IEM) [15–17].

It has been well established that the flux of protein import through TOC–TIC can vary greatly according to the developmental stage, environmental cues, or stress conditions [18–21]. The import process needs to be adequately regulated to maintain an optimally functioning chloroplast proteome [22]. The cytosolic ubiquitin-proteasome system (UPS) has emerged recently as an important regulator of chloroplast protein import [23]. The UPS is a eukaryotic protein-degrading mechanism that is involved in numerous protein homeostasis processes in the cell [24]. As a bacterially derived organelle, the chloroplast apparently contains no internal UPS machinery. Instead, ∼20 proteases of prokaryotic origin, notably Clp and FtsH enzymes, act to maintain internal protein homeostasis [18]. Alternatively, under conditions of stress or senescence, the entirety, or part, of the organelle may be degraded by autophagy [25–28]. However, autophagy and degradation by internal proteases are beyond the scope of this review, as indeed is the routing of proteins inside the chloroplast. Here, we will focus on the major events in the cytosol and at the chloroplast envelope membranes. The TOC–TIC system has been extensively reviewed elsewhere [2,6–8,29], and so our aim here is to summarize concisely recent advances. We endeavour to provide an overview of the mechanisms and components of the TOC–TIC apparatus, and to describe the several pathways through which protein import is regulated by the UPS.

## Protein targeting to the chloroplast surface

**Figure 1.**
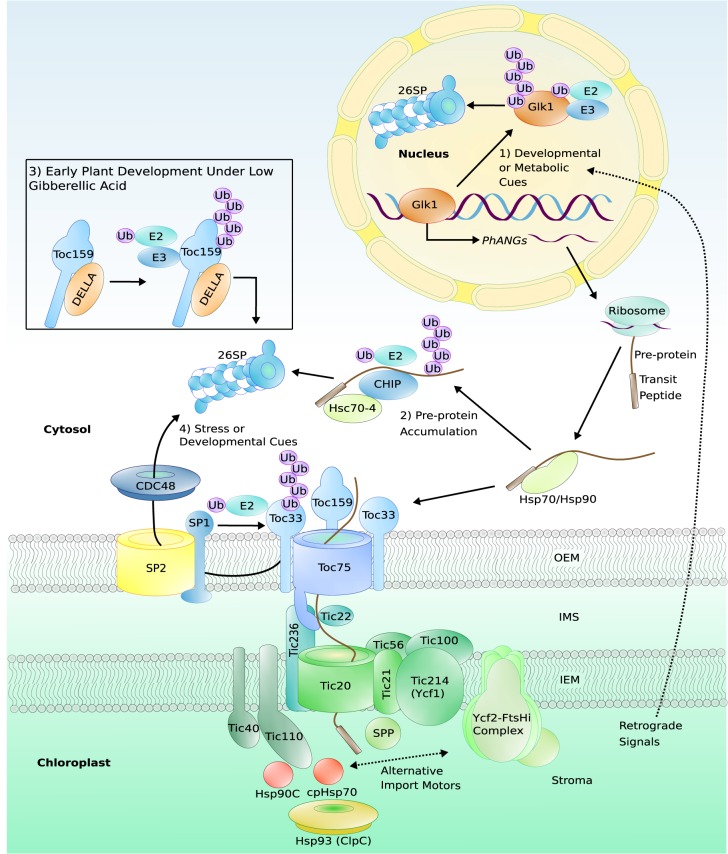
An overview of protein import into the chloroplast and its regulation by the UPS. Nucleus-encoded chloroplast proteins are synthesized in the cytosol as pre-proteins and post-translationally imported into the chloroplast. The pre-protein carries an N-terminal transit peptide which holds guidance information and initially allows interaction with cytosolic chaperones (e.g. Hsp70, Hsp90). The pre-protein is then recognized by receptor GTPases in the OEM, Toc33 and Toc159 (which exist in different isoforms with varying client specificity, and in *Arabidopsis,* these are termed Toc34 and Toc132/-120/-90, respectively). The receptors heterodimerize to allow the pre-protein to pass through the Toc75 pore into the IMS. Passage through the IEM is mediated by Tic20, which is reported to be part of the 1 MDa TIC complex containing Tic21, Tic56, Tic100, and Tic214 (Ycf1). Completion of translocation into the stroma is powered by an ATP-dependent import motor, which may be composed of stromal molecular chaperones (e.g. cpHsp70, Hsp90C) or a 2 MDa Ycf2–FtsHi complex. The Tic40 and Tic110 proteins are also involved in the import process, and may operate downstream in conjunction with stromal chaperones. Hsp93 (ClpC) has been proposed to perform a quality-control function at the point of import, or to act in the import motor. Upon arrival in the stroma, the transit peptide is cleaved from the pre-protein by the SPP. The UPS regulates protein import in a variety of ways: (1) The transcription factor Glk1, which regulates the expression of pre-protein-encoding PhaNGs, may be degraded by the UPS in response to unknown retrograde signals (from the chloroplast to the nucleus) that report on developmental or metabolic cues. (2) Accumulation of pre-proteins in the cytosol may trigger their UPS degradation to prevent the formation of cytotoxic aggregates, and this is mediated by the chaperone Hsc70-4 and the E3 ligase CHIP. (3) Before germination, DELLA factors repress chloroplast biogenesis under low gibberellic acid conditions by binding to Toc159 and triggering its UPS degradation. (4) During stress or particular phases of development, the CHLORAD system directly targets the TOC apparatus for proteolysis, with ubiquitination being mediated by the E3 ligase SP1. The targeted TOC proteins are retrotranslocated from the membrane via the channel protein SP2, using motive force provided by the cytosolic AAA+ ATPase CDC48. Note that the Toc33 and Toc159 receptor isoforms are depicted in the model here due to the known role of CHLORAD in suppressing the import of photosynthetic pre-proteins in response to abiotic stress [112], and because such photosynthetic pre-proteins are the primary clients of these isoforms [2]; however, all of the TOC receptor isoforms are likely substrates of SP1 at some stage, as revealed for example by the analysis of *sp1* plants during de-etiolation [109]. Dashed lines indicate uncertainty. Abbreviations: E2, E2 conjugase; E3, E3 ligase; 26SP, 26S proteasome; Ub, ubiquitin; SP, suppressor of *ppi1*; CDC48, cell division cycle 48; TOC, translocon at the outer envelope membrane of chloroplasts; TIC, translocon at the inner envelope membrane of chloroplasts; OEM, outer envelope membrane; IMS, intermembrane space; IEM, inner envelope membrane; SPP, stromal processing peptidase; GLK1, Golden2-like1; CHIP, C-terminus of Hsc70-interacting protein; PhANGs, photosynthesis-associated nuclear genes.

Chloroplast TPs possess remarkably diverse amino acid sequences. This may reflect their need to accommodate varying domains for interaction with cytosolic, chloroplast membrane, and stromal chaperones and sorting factors [30], or with different plastid types [31]. Cytosolic chaperones may facilitate the navigation of pre-proteins to the organelle and maintain an unfolded conformation suitable for import [32]. Two cytosolic systems are reported to guide pre-proteins, though their mechanistic details and physiological significance both require clarification. Hsp90 has been proposed to operate with Hsp70/Hsp90-organizing protein (Hop) and the immunophilin FKBP73 to deliver pre-proteins to the outer envelope membrane (OEM) (Figure 1), where it may dock at Toc64 [33,34]; however, such delivery to Toc64 may not be necessary for protein import [35,36]. Alternatively, Hsp70 (Figure 1) has been shown to act with an undefined 14-3-3 protein to recognize phosphorylated TPs and deliver them to translocation complexes [37]; however, mutation of the relevant phosphorylation site did not impair chloroplast targeting [38].

Apart from the canonical, TP-mediated TOC–TIC import route, there are several further targeting pathways that deliver chloroplast proteins; these are less well understood and thought to serve only several hundred proteins [2]. Proteins of the OEM typically do not possess a TP, with targeting information instead residing within a transmembrane domain [39,40]. An interesting exception is the β-barrel protein Toc75, which is inserted with the aid of a bipartite targeting peptide, the first part of which is a canonical TP [41,42]. It has recently emerged that other β-barrel proteins may insert with the aid of a TP [43], and those that do not may be targeted to the chloroplast by their penultimate β-barrel strand [44] via components of the TOC apparatus [43]. Lastly, there exist at least two non-canonical pathways of chloroplast protein targeting, which deliver internal proteins lacking an N-terminal signal in TOC-independent fashion [45], or involve passage through the endomembrane system [46].

## Protein import

### The outer envelope membrane

In the TOC complex, Toc33 and Toc159 are receptors that possess cytosol-projecting GTPase domains that bind to the TPs of pre-proteins [2]. In higher plants, both receptors are encoded by gene families and so exist in multiple isoforms (the associated nomenclature is based on their molecular masses in kilodaltons). Members of the Toc33 family have a relatively simple architecture, with a single C-terminal membrane-spanning domain [47] and an N-terminal GTPase domain [8]. Members of the Toc159 family share a similar GTPase domain [48], located centrally, and have a large C-terminal membrane-anchoring domain [8]. Toc159 isoforms typically also have an N-terminal, intrinsically disordered acidic domain, which may act to convey recognition specificity [49,50].

Pre-proteins are translocated through the OEM via a channel made by the β-barrel protein Toc75 (Figure 1). Toc75 belongs to the bacterially-descended Omp85 protein superfamily [51]. A feature of this family is a soluble N-terminal polypeptide transport-associated (POTRA) domain [52], which in the case of Toc75 extends into the intermembrane space (IMS) and performs a proposed chaperone-like role [53]. The Toc75 channel has been found to reach a maximum diameter of 30 Å [54]. The stoichiometry of the Toc33 : Toc75 : Toc159 core complex has been determined to be in the range of 4 : 4 : 1 [55] to 3 : 3 : 1 [56], and so the channel may be formed by multiple copies of the Toc75 protein [57].

Initial TP interaction with the GTPase domains of Toc159 and Toc33 is transient and energy independent [58], potentially allowing for rapid and sequential interaction with pre-proteins [59]. Later, the TP interacts with the POTRA domain of Toc75 and the soluble IMS protein Tic22, before GTP hydrolysis [59]. While GTP hydrolysis at both receptors is not necessary for protein import *in vivo* [60,61], it is required for successful protein translocation *in vitro* [59], reinforcing the notion that the GTPase receptors are the first points of contact [7,8,62]. The TP may bind simultaneously to Toc33 and Toc159, as each preferentially binds to a distinct region of the peptide [63]. A bound TP may then encourage heterodimer formation between Toc33 and Toc159, as well as GTP hydrolysis [64], leading to an activated translocon conformation which the pre-protein can pass through [8].

### The intermembrane space

In the IMS, Tic22 is suggested to act as a chaperone [65,66] and facilitate pre-protein delivery to the TIC complex at the IEM [67] (Figure 1). A recent study identified the IEM protein Tic236 as part of a 1.25 MDa TOC–TIC supercomplex [68]. Tic236 was suggested to provide a physical link between the TOC and TIC complexes, through its anchorage in the IEM, where it associates with Tic20, and its interaction with the POTRA domain of Toc75 [68] (Figure 1). However, a study of its maize orthologue, defective kernel5 (DEK5), identified functions in envelope biogenesis [69]. DEK5 was suggested to mediate the insertion of β-barrel proteins involved in protein import and metabolite transport, in accordance with the fact that it shares homology with the bacterial TamB protein [69]. A distinct role for Tic236 in protein import was proposed based on the observation that *tic236* mutant chloroplasts show reduced protein import but no change in the abundance of TOC proteins [68]. In contrast, *dek5* mutants displayed a reduction in TOC protein abundance, and thus the impact on protein import was interpreted to be a secondary effect [69]. Ultrastructure analysis revealed a reduction in the proportion of envelope relative to other chloroplast compartments in *dek5* [69], but a similar analysis was not done for *tic236* plants.

### The inner envelope membrane

The recent discovery of a 1 MDa TIC complex [70] has led to reconsideration of previously proposed models for the operation of the TIC machinery [6]. The 1 MDa complex consists of Tic214 (encoded by the chloroplast gene *ycf1*), Tic100, Tic56, Tic20, and Tic21 [70] (Figure 1). It is expected that the translocon channel is formed by Tic20, which has four membrane-spanning α-helical domains [71]. Three copies of Tic20 could theoretically exist within the 1 MDa complex [70], so a pore size of up to 30 Å has been predicted [57]. Tic21 has a similar structure to Tic20 and may function in a complementary way [72].

The originally identified components of the TIC import machinery include the proteins Tic110, Tic40, Tic20, and Tic21 (Figure 1). The functional relationship between Tic110 and Tic40 and the 1 MDa complex is unclear, but it may be that the former are recruited to import complexes during later stages of import to co-ordinate chaperone functions [6] and/or are required for the import of only some pre-proteins [73]. Stromal chaperones co-operate with Tic110 and Tic40 to facilitate pre-protein import and subsequent folding [74,75]. Translocation into the stroma is driven by an energy-dependent process [76] and several chaperones, including cpHsp70, Hsp90C, and Hsp93, have been implicated in the provision of the motive force [8] (Figure 1). However, the exact roles of these chaperones require clarification. While cpHsp70 has been strongly linked to the role of the main protein import motor [77–79], Hsp90C was also found to be essential for protein translocation [80]. A motor function has also been proposed for Hsp93 (ClpC) [81,82], although recent data showing that it interacts with the ClpP proteolytic subunit at the envelope (an interaction long known to occur in other contexts [83]) imply that it works in a protein quality-control process at the point of import [84,85].

The situation was further complicated by the recent identification of a 2 MDa Ycf2–FtsHi protein complex that was proposed to act as an import motor associated with the 1 MDa TIC complex [86] (Figure 1). In view of the evidence supporting 30 Å pore diameters for the import channels [57], it was suggested that such a powerful ATPase might be specifically recruited to handle the import of recalcitrant or tightly-folded proteins [87]. It seems plausible that cpHsp70 is the general import motor [77–79], with the energetically more expensive Ycf2–FtsHi complex being utilized only under specific conditions.

The 1 MDa TIC complex remains an enigmatic discovery in need of further research. It was the first import complex to contain a chloroplast-encoded protein (Tic214/Ycf1), and this component is notably absent from the family Poaceae [2]. The complex has been proposed to act as a general TIC translocon at the IEM, and in support of this notion, it co-purifies with TOC proteins [70], an interaction that could in principle be mediated by Tic236. Knock-out mutants of *ycf1* are embryo lethal, and *tic100* and *tic56* mutants display chlorotic phenotypes [70] typical of impaired chloroplast protein import. However, analysis of *tic56* mutants attributed the observed phenotypes to defects in ribosome assembly, and revealed no impairment of protein import [88–91], a result shared when *ycf1* translation was repressed by the specific plastid ribosomal inhibitor spectinomycin [92]. Tic214 has also been found to have functions in the biogenesis of photosynthetic complexes in thylakoid membranes [93], which may contribute to the severity of its knock-out phenotype. It is plausible that the 1 MDa TIC components act in multiple processes, including protein import, with additional machinery operating either in series or in parallel. The extent of involvement of the 1 MDa complex in import may depend on the nature of the client proteins, or on specific developmental or environmental conditions.

### Ubiquitin-dependent regulation of protein import

The UPS is a major regulatory system involved in the targeting of misfolded or unnecessary proteins for degradation. It functions within many biological processes, such as hormone signalling [94]. Ubiquitination (or ubiquitylation) is a post-translational modification involving the addition of one or more copies of the 8.5 kDa ubiquitin protein to lysine residues of target proteins [24]. The addition of polyubiquitin chains identifies the protein for degradation by the nucleocytosolic 26S proteasome (26SP) [24]. The 26SP is an ATP-dependent proteolytic complex formed from a cylindrical 20S core particle and a 19S regulatory particle; ubiquitinated proteins are recognized by the regulatory particle which guides them to the core where they are degraded [95]. Importantly, this cytosolic machinery also targets organelles, most famously the endoplasmic reticulum (ER) in ER-associated protein degradation (ERAD) [96], but also the endosymbiotically derived mitochondria and chloroplasts [97]. Indeed, proteomic studies of plant ubiquitinomes have identified many chloroplast proteins which may be UPS targets, although it remains to be determined whether these are processed as precursors in the cytosol or later [98–100].

Ubiquitination requires an enzyme cascade involving the activation and targeted conjugation of ubiquitin. An E1 ubiquitin activase first forms a thioester bond with ubiquitin in an ATP-dependent reaction [24]. The ubiquitin moiety is then transferred to an E2 ubiquitin conjugase. Finally, an E3 ubiquitin ligase conveys substrate specificity through a selective interaction with its targets, catalysing the transfer of ubiquitin to the target from the E2 enzyme [24]. Reiterative rounds of conjugation onto ubiquitin lysine residues result in the formation of a polyubiquitin chain degradation signal. Upon degradation, the ubiquitin moieties are recycled through the action of deubiquitinating enzymes. The E3 ligases are necessarily numerous and diverse, given their role in specificity, with roughly 1400 E3 proteins in *Arabidopsis*, far outnumbering the ∼40 E2 and two E1 enzymes [23]. In plants, there are four classes of E3 ligase: homologous to the E6-AP carboxyl terminus (HECT), really interesting new gene (RING), U-box, and cullin-RING ligase (CRL) [23]. Each class has a different mechanism of action and subunit composition, with particular diversity in the substrate-interacting domain or component [24].

### Degradation of precursor proteins

Initial evidence for the UPS regulation of chloroplast proteins came from observations of cytosolic E3 activity targeting pre-proteins [101]. The C-terminus of Hsc70-interacting protein (CHIP) E3 ligase was initially shown to direct the degradation of Clp and FtsH precursors under high-light conditions [102,103]. A subsequent study revealed that in the *Arabidopsis* Toc159 mutant *plastid protein import2* (*ppi2*), pre-proteins had accumulated in the cytosol and the expression of the cytosolic chaperone Hsc70-4 (an Hsp70 isoform) was elevated [101]. The Hsc70-4 protein was shown to interact with the TPs of pre-proteins, recruiting them to CHIP for ubiquitination and degradation by the 26SP [101] (Figure 1). This system is suggested to function as a quality-control process to degrade mis-targeted proteins and/or prevent the accumulation of pre-proteins in the cytosol, as unfolded proteins may accumulate into cytotoxic aggregates [101]. A recent study in wheat identified another cytosolic E3 ligase, stress-associated protein 5 (SAP5), which interacts with the pre-protein of Hsp90C to trigger its degradation [104].

### Chloroplast biogenesis

A further influence of the UPS on cytosolic events controlling chloroplast biogenesis was reported to occur during early plant development, pre-germination. DELLA proteins inhibit seed germination in processes regulated by UPS-mediated degradation [105]. Germination depends on the accumulation of the hormone gibberellic acid, which down-regulates DELLA factor accumulation to enable germination and, in turn, chloroplast biogenesis [106]. It was reported that all DELLA factors can interact with cytosolic Toc159, prior to its assembly into the TOC complex, and initiate its degradation by the 26SP [106] (Figure 1). Low gibberellic acid conditions also resulted in the UPS-dependent down-regulation of pre-proteins [106].

The UPS also exerts indirect effects on chloroplast development through nuclear activities [23,25,97,107]. In *Arabidopsis*, the two golden2-like (Glk) transcription factors function redundantly to promote the expression of photosynthetic proteins, thereby promoting chloroplast biogenesis [108]. Glk1 itself is regulated at the transcriptional level through plastid-to-nucleus signals mediated by genomes uncoupled1 (GUN1) in response to the developmental state of the organelle [9]. Interestingly, Glk1 has also been found to be regulated at the posttranslational level by the UPS in response to an as yet unknown, GUN1-independent plastid signal [108] (Figure 1). This signal may derive from an environmental or developmental source to control chloroplast biogenesis [108].

### Chloroplast-associated protein degradation

The first evidence for direct interaction between the UPS and chloroplast proteins *in situ* came from the discovery of the ubiquitin-dependent degradation of TOC complexes. A forward-genetic screen identified the RING-type E3 ligase, suppressor of *ppi1* locus 1 (SP1) [109]; *ppi1* is a Toc33 knock-out mutant with a pale yellow phenotype [110]. Located in the OEM [111], SP1 possesses two transmembrane domains separated by an IMS domain, which acts in target recognition, and a C-terminal cytosolic RING domain [109]. SP1 directly interacts with all TOC proteins, mediating their ubiquitination and degradation (Figure 1). Thus, there is an increase in TOC protein abundance when SP1 is lost, which, when in a chlorotic *ppi1* background, causes enhanced greening (or suppression of *ppi1*), a phenotype mirrored by UPS inhibition [109]. Further experiments revealed that such degradation of TOC complexes provides for important regulation of the import machinery, and may act to alter the proteome, functions and developmental fate of the organelle [112]. This regulation can also help to promote the plant's tolerance of abiotic stress, by attenuating the import of photosynthetic proteins and thus suppressing photosynthesis and the tendency to overproduce reactive oxygen species during stress [112].

To be degraded by the cytosolic 26SP, polyubiquitinated TOC proteins first need to overcome the physical and energetic barriers to their removal from the OEM. Degradation of ER membrane and mitochondrial outer membrane proteins involves retrotranslocation across the membrane before degradation by the 26SP [96,113], and it was thought that protein degradation at the chloroplast OEM may involve an analogous process. Strikingly, an additional product of the suppressor screen that identified SP1 was the Omp85 protein SP2, and this OEM protein was shown to assist TOC retrotranslocation [114]. As an Omp85 family member, SP2 shares homology with Toc75 and is capable of forming a channel, although it lacks a POTRA domain. Like SP1, SP2 physically interacts with TOC proteins, and it is hypothesized to form the retrotranslocon channel [114] (Figure 1). The entry of substrates into the channel may occur by lateral gating in the membrane, as with other Omp85 proteins [57].

The motive force for extraction through SP2 is provided by a cytosolic factor: cell division cycle 48 (CDC48) is a homohexameric ATPase and a member of the ATPases associated with various cellular activities (AAA+) family of proteins [115]. Conformational changes in CDC48 induced by ATP hydrolysis create a motive force, allowing for the extraction and denaturation of bound substrates through the central pore [116,117] (Figure 1). CDC48 functions in a variety of cellular activities beyond protein homeostasis, such as cell cycle regulation and autophagy, but it is especially well known as the core motor component in ERAD [118]. Of the five reported homologues of CDC48 in *Arabidopsis*, CDC48A was found to associate with Toc33 [114], and has recently been identified by proteomic analysis of the chloroplast envelope [119]. Reconstitution experiments demonstrated that CDC48 operates as a cytosolic motor to retrotranslocate ubiquitinated TOC proteins prior to their degradation, a process in which SP2 was also shown to be critical [114] (Figure 1). This pathway of TOC degradation by the UPS involving SP1, SP2, and CDC48 has been named CHLORAD [114].

## Perspectives

- *Importance of the field*: The chloroplast is a dynamic plant organelle with indispensable metabolic functions including photosynthesis. The import of chloroplast proteins is, therefore, an essential and tightly controlled process, defects in which result in severe phenotypes. The UPS-mediated regulation of chloroplast protein import enables the plant to respond to the fluctuating protein demands of the organelle.
- *Summary of current thinking*: Despite concerted efforts to unravel the mechanisms of protein import into chloroplasts over many years, there remain substantial gaps in our understanding. Passage through the TOC apparatus is a reasonably well-defined process involving initial docking at receptor GTPases and subsequent translocation through the β-barrel pore of Toc75. However, precisely how pre-proteins are delivered across the IEM is unclear: two TIC systems, involving Tic110/Tic40 and Tic214 (the 1 MDa complex), have been implicated, but whether and/or how they co-operate is unknown. Moreover, while it is well established that an ATP-dependent motor drives protein import, whether this comprises stromal chaperones (including cpHsp70) or a 2 MDa Ycf2–FtsHi complex is debated. The UPS is known to regulate the import process in a variety of ways: it degrades pre-proteins in the cytosol; it down-regulates chloroplast biogenesis in early development through the destruction of Toc159, and the transcription factor Glk1; and it reconfigures the TOC apparatus during developmental transitions and periods of stress.
- *Future directions*: It is clear that there is a need to reconcile the conflicting reports on the cytosolic guidance systems, the TIC apparatus, and the stromal import motor. With regard to UPS involvement in chloroplast protein import, our understanding is still in its infancy. It is apparent from proteomic studies that a greater number of chloroplast proteins may be regulated by the UPS than is currently appreciated. Processing of many of these additional proteins may occur through the degradation of pre-proteins in the cytosol, and so additional cytosolic E3 ligases may be identified. Moreover, how the UPS regulation of chloroplasts acts in a physiological context requires further elucidation: we have seen that pre-germination conditions promote pre-protein degradation, and that CHLORAD can mediate stress responses, and so it seems likely that additional integration with developmental and environmental cues will be revealed. The discovery of the CHLORAD pathway offers much promise for future studies. In addition to identifying additional components and cofactors of the system, it will be important to determine the full range of its substrates. As things stand, only TOC proteins (and SP1) are established CHLORAD substrates, but there does exist the potential for other substrates to be found, and indeed for the identification of additional E3 ligases. Lastly, how the activity of CHLORAD is co-ordinated by intracellular signalling networks is completely unknown, and this will be very interesting to explore.

## Abbreviations

26SP: 26S proteasome
AAA+: ATPases associated with various cellular activities
CDC48: cell division cycle 48
CHIP: C-terminus of Hsc70-interacting protein
CHLORAD: chloroplast-associated protein degradation
CRL: cullin-RING ligase
DEK5: defective kernel5
ER: endoplasmic reticulum
ERAD: ER-associated protein degradation
FKBP73: FK506-binding protein 73
Glk1: golden2-like 1
HECT: homologous to the E6-AP carboxyl terminus
Hop: Hsp70/Hsp90-organizing protein
IEM: inner envelope membrane
IMS: intermembrane space
OEM: outer envelope membrane
POTRA: polypeptide transport-associated
*ppi*: plastid protein import
RING: really interesting new gene
SP: suppressor of *ppi1*
TIC: translocon at the inner envelope membrane of chloroplasts
TOC: translocon at the outer envelope membrane of chloroplasts
TP: transit peptide
UPS: ubiquitin-proteasome system
